# Colistin Resistance in Gram-Negative Bacteria: Mechanisms, Transmission, and Novel Intervention Strategies

**DOI:** 10.3390/microorganisms14010173

**Published:** 2026-01-13

**Authors:** Shah Zeb, Arzoo Nazir, Muhammad Fazal Hameed, Sadia Ikram, Syed Zeeshan Haider Naqvi, Muhammad Shoaib, Patrick Butaye, Zhiqiang Wang, Ruichao Li, Xiaoyu Lu

**Affiliations:** 1 Jiangsu Co-Innovation Center for Prevention and Control of Important Animal Infectious Diseases and Zoonoses, College of Veterinary Medicine, Yangzhou University, Yangzhou 225009, China; shahzeb1267@gmail.com (S.Z.); hamthemicrobiologist@gmail.com (M.F.H.); zqwang@yzu.edu.cn (Z.W.); rchl88@yzu.edu.cn (R.L.); 2 Institute of Comparative Medicine, College of Veterinary Medicine, Yangzhou University, Yangzhou 225009, China; arzoonazir68@gmail.com; 3 Institute of Molecular Biology and Biotechnology (IMBB), The University of Lahore, Lahore 54000, Pakistan; drsadiaikram79@gmail.com (S.I.); zeeshan.haider@imbb.uol.edu.pk (S.Z.H.N.); 4 Department of Pathology, HBS Medical and Dental College, Islamabad 44000, Pakistan; 5 Department of Infectious Diseases and Public Health, Jockey Club College of Veterinary Medicine and Life Sciences, City University of Hong Kong, Kowloon, Hong Kong, China; pabutaye@cityu.edu.hk; 6 College of Pharmacy, Taizhou University, Taizhou 225300, China

**Keywords:** colistin resistance, Gram-negative bacteria, One Health approach, *mcr* genes, intervention strategies

## Abstract

Multidrug resistance (MDR) in Gram-negative bacteria is a global issue and needs to be addressed urgently. MDR can emerge through genetic mutations and horizontal gene transfer and deteriorate under antibiotic selective pressure. The emergence of resistance to last-resort antibiotics, which are used to treat MDR bacteria, is of particular concern. Colistin has been recognized as a last-line antibiotic for the treatment of MDR Gram-negative bacterial infections caused by *Escherichia coli*, *Acinetobacter baumannii*, *Klebsiella pneumoniae*, and *Pseudomonas aeruginosa*. Recently, the increasing reports of colistin resistance pose a significant threat to public health, caused by both acquired and intrinsic mechanisms. The review aimed to elucidate the trends in colistin resistance, the use of colistin in human and veterinary medicine, underlying resistance mechanisms and transmission pathways, and potential mitigation of this emerging threat through novel intervention strategies. Colistin resistance is mediated by plasmid-encoded phosphoethanolamine transferases (*mcr-1* to *mcr-10*) and chromosomal lipid A remodeling pathways. In *Escherichia coli*, resistance involves *mcr-1–10*, *acrB* efflux mutations, *pmrA/pmrB*, *arnBCADTEF*, and *mgrB* inactivation. *Klebsiella pneumoniae* exhibits *mcr-1*, *mcr-8*, *mcr-9*, *mgrB* disruption and *phoP/phoQ–pmrAB* activation. *Acinetobacter baumannii* harbors *mcr-1–4*, while *Salmonella enterica* and *Enterobacter* spp. carry *mcr* variants with *arnBCADTEF* induction. Therapeutic options include adjunct strategies such as antimicrobial peptides, nanomaterials, therapeutic adjuvants, *CRISPR-Cas9*-based gene editing, probiotics, vaccines, and immune modulators to restore susceptibility. This review identified that specific and wide actions are required to handle the growing colistin resistance, including genomic surveillance, tracing novel resistance mechanisms, and the application of alternative management strategies. The One Health approach is considered a key strategy to address this growing issue.

## 1. Introduction

Antibiotic resistance in Gram-negative bacteria poses a significant threat, as limited treatment options are available. An increase in the consumption of antibiotics makes bacteria more prone to be resistant to treatment [1,2]. It results in antibiotic resistance and transmission through plasmids within One Health settings that undermine the health of humans, animals, and the environment [3,4]. Healthcare settings are dependent on antibiotics to treat and prevent infections arising from usual medical treatments. Combinations such as ceftolozane–tazobactum, ceftazidime–avibactam, and meropenem–vaborbactum are employed in the fight against significant resistant strains, such as *Acinetobacter baumannii*, *Pseudomonas aeruginosa*, and *Enterobacteriaceae*, particularly those that produce metallo-beta-lactamases (MBLs), like New-Delhi MBL (NDM) [5,6]. Due to the high level of antibiotic resistance in multidrug-resistant (MDR) pathogens, such as the ESKAPE group in the healthcare and veterinary settings, the world is facing a serious challenge [7,8,9].

Colistin is an antimicrobial agent that belongs to the polymyxin class of antibiotics derived from *Paenibacillus polymyxa*. Five polymyxins make up this class: A, B, C, D, and E. Of these, polymyxins B and E (colistin) are clinically important [10]. In human medicine, parenteral therapy is administered using colistin methanesulfonate (CMS) sodium, while oral and topical treatments are handled with colistin sulfate (CS). Colistin is among the last-choice antibiotics that are widely used for the treatment of drug-resistant bacterial infections [11,12]. It is also used as a protective and growth agent in veterinary medicine. It has been more well-known in recent years as a last-choice therapy for infections caused by Gram-negative bacteria that are resistant to most drugs; these infections typically involve Enterobacterales, *Pseudomonas aeruginosa*, and *Acinetobacter baumannii*, which produce carbapenemase [13].

**Figure 1:**
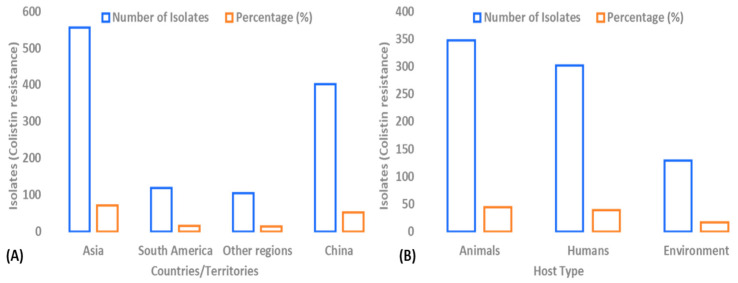
Trends of colistin resistance in One Health. (**A**) Geographical representation of colistin resistance (number and percentage) in different regions and China. (**B**) Host-based representation of colistin resistance (number and percentage) in animal, human, and environmental sources.

Colistin resistance has become a global concern (Figure 1), as colistin is frequently used as a last-resort antibiotic to treat MDR Gram-negative bacterial infections. Frequent use of colistin in veterinary practices has resulted in wide spread of colistin resistance and is promoted by the mobilized colistin resistance gene *mcr-1* along with its variants [14,15]. These genes keep on regulating within the bacterial community, causing growing resistance problems. Not only *mcr* but also some other acquired and intrinsic genes contribute to colistin resistance. One hot topic of antimicrobial resistance is to find out the mechanisms behind colistin resistance [16]. To learn more about the processes underlying colistin resistance, researchers are utilizing modern genomics techniques, such as whole-genome sequencing, in conjunction with molecular biology, proteomics, and bioinformatics methods. To tackle this antimicrobial resistance (AMR) challenge, different strategies have been developed, including the invention of new drugs, re-purposing previous ones, the employment of nano-based approaches, combining colistin with other treatments, photodynamic therapy, phage-based therapy, and CRISPRi-based strategies with other therapies. [17]. This review aimed to highlight the role of colistin in human and veterinary medicine and how it plays its role in the spread of AMR. It also focused on the role of the “One Health approach” to manage the usage of antibiotics across different areas, preventing the continued widespread resistance to colistin. It also aimed to summarize novel techniques and strategies to tackle resistance against colistin.

## 2. Methodology

In the current review, data were systematically retrieved from key scholarly databases such as PubMed, Scopus, and Web of Science using predefined search algorithms and keywords, including “colistin resistance” OR “polymyxin resistance,” “colistin resistance in Gram-negative bacteria,” and “colistin resistance in One Health”. Moreover, the studies published in peer-reviewed journals were included to strengthen the study objectives. The selection criteria focused on relevance to the central themes and arguments developed in the narrative review, such as Theme A: colistin resistance mechanisms, Theme B: possible transmission pathways for colistin resistance spread, Theme C: emerging technologies in diagnosing colistin resistance, and Theme D: emerging strategies to tackle colistin resistance. Studies were selected for their ability to illustrate, critique, or advance discussion on these themes.

## 3. Mechanism of Colistin Resistance in Gram-Negative Bacteria

Colistin is a last-resort antibiotic that targets Gram-negative bacteria by binding to lipopolysaccharides (LPSs) in the outer membrane, displacing stabilizing cations, such as magnesium (Mg^2+^) and calcium (Ca^2+^), and disrupting membrane integrity. Bacterial resistance to colistin emerges primarily through modifications of lipid A, the lipid anchor of LPSs, which reduces colistin’s binding affinity. These modifications are mediated by both chromosomal regulatory systems and plasmid-borne resistance genes. The *PhoPQ* two-component system plays a central role in colistin resistance. *PhoQ*, a membrane-bound sensor kinase, is activated under conditions of low Mg^2+^, acidic pH, or the presence of colistin itself. Once activated, *PhoQ* phosphorylates its cognate regulator *PhoP*, which induces transcription of genes, such as *pmrD* and the *arnBCADTEF* operon. The *arnBCADTEF* operon encodes enzymes responsible for the addition of 4-amino-4-deoxy-L-arabinose (L-Ara4N) to lipid A, decreasing its negative charge and thereby reducing colistin binding. Importantly, *PhoP* also activates *pmrD*, which stabilizes the activity of the *PmrAB* system, creating cross-regulation between the two pathways [18].

**Figure 2:**
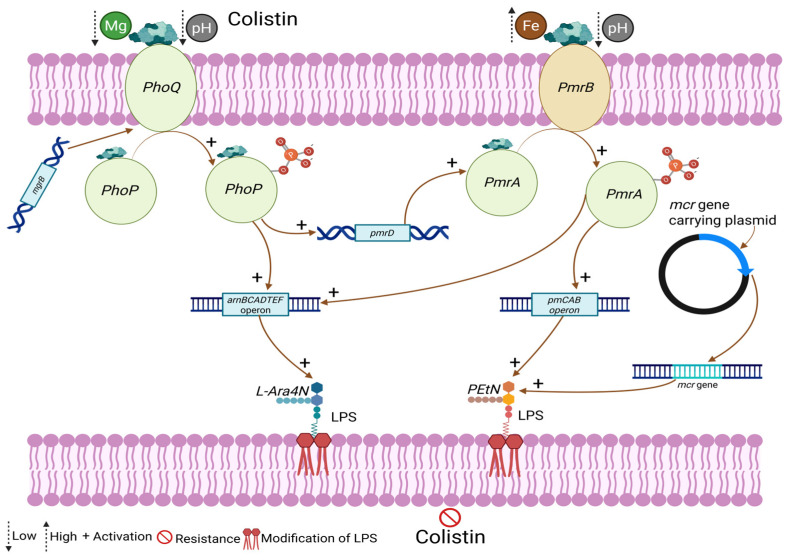
Schematic representation of PhoPQ and PmrAB two-component systems and plasmid-borne *mcr* genes contributing to colistin resistance in Gram-negative bacteria. Environmental signals such as low Mg^2+^ or acidic pH activate the sensor kinase *PhoQ*, leading to phosphorylation of *PhoP*, which induces expression of the *arnBCADTEF* operon and *pmrD*. PmrD links *PhoPQ* to the *PmrAB* system by stabilizing phosphorylated PmrA. Independently, PmrB is activated by stimuli such as Fe^3+^ or low pH and phosphorylates PmrA, which directly upregulates the *pmrCAB* operon. The *arnBCADTEF* operon mediates the addition of *L-Ara4N*, while the *pmrCAB* operon mediates *PEtN* addition to LPS. Plasmid-encoded *mcr* genes independently catalyze *PEtN* modification of lipid A. These LPS modifications reduce the net negative charge of the outer membrane, decreasing colistin binding and resulting in colistin resistance. Symbols indicate activation (+), signal intensity (low to high), LPS modification, and resistance outcome. (Created in https://BioRender.com, accessed on 13 May 2025).

The *PmrAB* two-component system is activated by high iron (Fe^3+^) concentrations or acidic pH. PmrB, the sensor kinase, phosphorylates the response regulator *PmrA*, which then induces the *pmrCAB* operon. This operon encodes a phosphoethanolamine (*PEtN*) transferase that attaches *PEtN* to lipid A. Like *L-Ara4N* modification, the addition of *PEtN* neutralizes negative charges on lipid A, further reducing colistin’s ability to bind. Thus, the *PhoPQ* and *PmrAB* systems work together to remodel the bacterial outer membrane and confer resistance [19]. In addition to these chromosomal mechanisms, resistance can be acquired via plasmid-borne mobilized colistin resistance (*mcr*) genes. These genes encode *PEtN* transferases similar to those encoded by *pmrCAB*, enabling bacteria to modify lipid A with *PEtN*. The plasmid-mediated nature of mcr genes allows horizontal transfer between bacterial species, greatly amplifying the threat of colistin resistance. Together, these mechanisms—whether through *PhoPQ*-regulated *L-Ara4N* addition, *PmrAB*-regulated *PEtN* modification, or plasmid-borne *mcr* genes—confer protection against colistin and compromise its effectiveness as a last-line antibiotic, as shown in Figure 2 [20].

## 4. *mcr* Genes and Associated Resistance Pathways in Gram-Negative Bacteria

**Figure 3:**
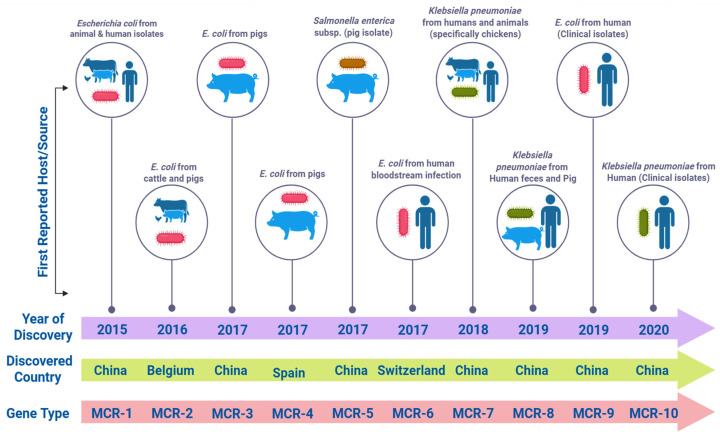
Chronological discovery and spread of *mcr-1* to *mcr-10* plasmid-mediated colistin resistance genes (created in https://BioRender.com, accessed on 15 May 2025).

In most cases, the colistin resistance mechanism has been encoded by chromosomes, and still, a single transferable mechanism of resistance was identified [21]. After the discovery of *mcr-1* in China, ten *mcr* genes (*mcr-1* to *mcr-10*) and a number of variants have been discovered and identified, as shown in Figure 3 [22]. Liu et al. were the first to discover the plasmid-mediated mobilized colistin resistance gene *mcr-1* in humans and animals in *Enterobacteriaceae* [23]. The PEtN transferase enzyme is encoded by the *mcr* gene, which results in the modification of lipid A by adding the PEtN group at the 4′-phosphate positions, causing colistin resistance. This resistance is mediated by changes in LPSs, due to which the overall charge of LPS changes, ultimately reducing the binding attraction of polymyxins with the outer membrane of bacteria [24].

In the *Enterobacteriaceae* family, resistance to polymyxins in different genera, including *Enterobacter*, *Klebsiella*, *Salmonella*, and *Escherichia*, has been reported [25]. As with naturally occurring colistin-resistant strains, the addition of cationic groups, including *L-Ara4N* and phosphoethanolamine (*PEtN*), to the LPS is thought to be the cause of Enterobacteriaceae’s colistin resistance. The resistance seemed to arise from modifications occurring in the LPS through operon and chromosomal genes encoding enzymes involved in LPS modification, like *pmrE* and *pmrC* genes, and the *pmrHFIJKLM* operon [26]. A significant role in the regulation of these modifications has been played by regulatory two-component systems (TCSs), like *crrA-crrB*, *PhoP-PhoQ*, and *PmrA-PmrB*, along with *mgrB*, which act as a negative regulator of *PhoP-PhoQ*. Along with this, resistance is also caused by plasmid-mediated *mcr* genes along with the *rcs* and *cox* systems by upregulating capsule biosynthesis, regulating the *PhoP-PhoQ* system, and activating efflux pumps [27], as shown in Table 1.

Another potential cause of colistin resistance has been found to be disruptions or mutations in the *mgrB* gene [28]. Inactivation of *mgrB* has been found to be a significant mechanism of resistance in *Klebsiella oxytoca* and *Klebsiella pneumoniae*. Another cause of colistin resistance development was reported to be amino acid substitutions in the CrrB protein that can lead to a rise in autophosphorylation [29].

The One Health approach identifies the ecological and evolutionary imperatives for colistin resistance [30]. The global use of colistin in agriculture, especially in intensification systems for livestock production, has introduced selective pressure conducive to the persistence and transmission of *mcr*-mediated resistance throughout bacterial populations in animals, soil, and aquatic environments [31]. These resistant bacteria and mobile genetic entities have been introduced to humans through direct contact with animals, food sources, and exposure to the environment, establishing a complicated transmission cycle. Significantly, these environmental reservoirs, like wastewater treatment systems and surface waters, are foci for the exchange of resistance genes, raising the probability of reintroduction into clinical as well as agricultural environments [32]. These avenues can only be addressed through integrated interventions: investing in rapid diagnostic tools, enhancing wastewater treatment, and encouraging global cooperation [33]. Such a multi-focused approach demonstrates how the approach applied through One Health can link human, animal, and environmental health to limit the worldwide transmission of colistin resistance [34].

**Table 1:** Mechanisms of colistin resistance in Gram-negative bacterial species with a focus on *mcr* genes and associated resistance pathways.

Bacteria	Genetic Factor	Associated Resistance Pathways	References
*Salmonellaenterica*	*mcr-1*, -*3*, and -*9*	Phospohoethanolamine transferase	[35,36]
	*arnBCADTEF*	Phospohoethanolamine transferase modification of lipid A	
*Aeromonas* species	*mcr-1*, -*3*, and -*5*	Phospohoethanolamine transferase	[37]
*Escherichia coli*	*mcr-1* to *mcr-10*	Phosphoethanolamine transferase	[23,38]
	*acrB* mutation	Efflux pump	
	*arnBCADTEF*	Lipid A modification by *L-4AraN*	
	*pmrB*/*pmrA*	Lipid A modification by *pmrE* and *pmrC* genes and *arnBCADTEF*	
	*mgrB* mutation	Activation of *pmrHFIJKLM* and *phoPQ* overexpression	
*Pseudomonas aeruginosa*	*PhoPQ*, *pmrAB*	Low Zn^+2^ in the addition of LPSs	[39]
	*mcr-1* and -*2*	Phosphoethanolamine transferase	
*Vibrio cholerae*	*IpxN* and *gspIEF*	Modifications in the LPS moiety	[40]
*Acinetobacter baumannii*	*mcr-1*, -*2*, -*3*, and -*4*	Phosphoethanolamine transferase	[41]
*Klebsiella pneumoniae*	*PhoQ*/*phoP*	The activation of *pmrAB* by *pmrD* or the activation of the *pmrHFIJKLM* operon results in the modification of lipid A	[42]
	*ramA*	Modulates lipid A biosynthesis	
	*mcr-1*, -*8*, and -*9*	Phosphoethanolamine transferase	
	*mgrB* mutation	Activation of *pmrHFIJKLM* and overexpression of *phoPQ*	[43]
*Enterobacter* species	*phoQ*/*phoP*	4-amino-4-deoxy-L-arabinose and Phospohoethanolamine transferase result in the modification of lipid A	[44,45]
	*mcr-1*, -*4*, -*5*, and -*10*	Phosphoethanolamine transferase	
	*arnBCADTEF*	Activation of *pmrHFIJKLM* and overexpression of *phoPQ*	

### 4.1. mcr-Driven Colistin Resistance

In the microbial world, the leading causes of bacterial resistance to colistin are plasmids that are transposable genetic elements carrying the *mcr* genes, as shown in Figure 2 [16]. Up to now, around ten variants of mobilized colistin resistance genes *mcr-1* to *mcr-10* have been known. Several chromosomal genes, including *PhoP-PhoQ*, *mgrB*, and *PmrA-PmrB* (two-component regulatory systems), biofilm formation, mutations, and efflux pumps, have been linked to the development of colistin resistance [25]. Moreover, *mcr* is the primary factor responsible for plasmid-borne colistin resistance. Numerous molecular mechanisms have been proposed to explain this resistance; however, the mechanisms of colistin resistance are not understood fully for some bacterial species, and our understandings remain incomplete [46].

These *mcr*-containing bacteria have been found in humans, poultry, the environment, and animals across six continents of the world [47,48]. The *Escherichia coli* species has been identified as positive with several *mcr* genes, each associated with the *PhoP-PhoQ* regulatory system. For the *mcr-1* gene, the length of 1626 base pairs (bp) was amplified in human, animal, and food samples from China [49]. For *mcr-2*, a 1617 bp gene was isolated from animals in Germany [50]. Animal samples from China have also identified the 1626 bp *mcr-3* gene. For the *mcr-4* gene, a 1626 bp gene has been recently amplified both in *Escherichia coli* and *Salmonella* spp. These were also from animal sources from Spain, Germany, and Italy [25]. The *mcr-5* gene was found to be linked with *Salmonella* spp. with a 1644 bp, which similarly reported its isolate in foods and animals from Germany [51]. A novel *mcr* variant, *mcr-6* with a 1617 bp, was amplified from animal-derived *Moraxella pluranimalium* in the United Kingdom [52]. The *mcr-7* gene (1620 bp) and the *mcr-8* gene (the size not determined) have been detected in *Klebsiella pneumoniae* isolates recovered from animals in China. In addition, the *mcr-9* gene (the size not determined) has been identified in *Salmonella typhimurium* isolates recovered from humans in the United States [53]. The *mcr-10* gene, considerably longer at 1775 bp, was found in *Escherichia coli* from chicken samples in China [54]. The *mcr-10* gene was found to be situated on the IncFIA(HI1) plasmid, underlining its connection with mobile genetic elements that facilitate horizontal gene transfer. In China, recently, *mcr-10* was identified from a clinical *Enterobacter roggenkampii* strain, which had a non-conjugative IncFIA(HI1) plasmid, and 80% nucleotide of *mcr-10* was associated with *mcr-9*, which increased colistin minimum inhibitory concentration (MIC) fourfold from 1 to 4 mg/L [55].

The *mcr-1* gene remains the most widely distributed *mcr* variant, having been reported in over 50 countries and regions across six continents [31]. It has been identified in multiple bacterial genera, including *Escherichia coli*, *Klebsiella pneumoniae*, *Salmonella*, *Shigella*, *Pseudomonas aeruginosa*, *Acinetobacter baumannii*, *Citrobacter braakii*, *Enterobacter sakazakii*, and *Raoultella planticola*, with *Escherichia coli* serving as the primary reservoir of *mcr-1* [31,56]. Currently, *mcr-1*-positive *Escherichia coli* strains are widely distributed among animal sources, including food-producing animals, companion animals, wildlife, insects, and so on [57], while also being frequently detected in humans (both clinical and healthy populations) and environmental samples [58]. To investigate the global prevalence of *mcr*-positive *Escherichia coli*, Caldes et al. conducted a comprehensive analysis of 22,884 *Escherichia coli* strains available in the NCBI database, identifying 778 *mcr*-positive isolates [56]. These 778 *mcr*-positive *Escherichia coli* strains spanned 25 countries across six continents, with the majority concentrated in Asia (555/778, 71.33%) and South America (119/778, 15.30%). China showed the highest prevalence (401/778, 51.54%). The strains were widely distributed among animals (347/778, 44.60%), humans (302/778, 38.82%), and environmental sources (129/778, 16.58%). Among animal reservoirs, chickens (202/347, 58.21%), pigs (44/347, 12.68%), and cattle (32/347, 9.22%) were the most common hosts. These 778 *mcr*-positive *Escherichia coli* strains exhibited significant diversity, belonging to 239 distinct sequence types (STs), with ST10 (107/778, 13.75%) being the most prevalent, followed by ST156 (46/778, 5.91%) and ST48 (29/778, 3.73%). The *mcr-1* variant was the dominant genotype (654/778, 84.06%) [59,60,61].

### 4.2. PmrB Mutations Lead to Colistin Resistance in *Acinetobacter baumannii*

**Figure 4:**
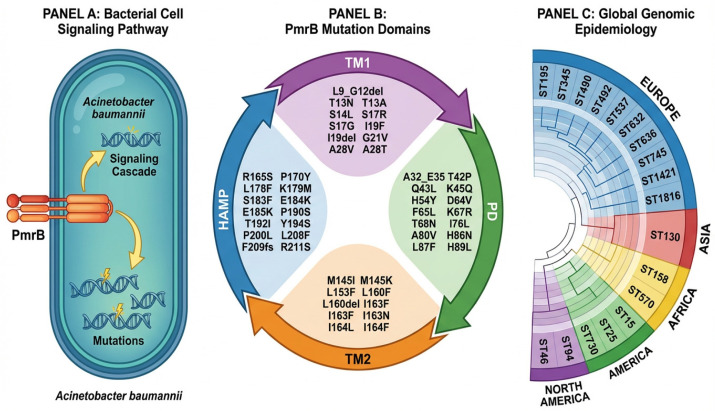
(**A**) Colistin resistance in *Acinetobacter baumannii* is primarily mediated by mutations in the PmrB sensor kinase protein, a key component of the PhoP-PhoQ regulatory system. (**B**) Specific amino acid mutations in each domain are detailed, showing diverse substitutions that contribute to resistance phenotypes. The mutations span critical domains involved in signal transduction and regulatory functions of the PmrB protein. (**C**) *Acinetobacter baumannii* sequence types (STs) identified in Europe, Africa, North America, and South America (created in https://BioRender.com accessed on 20 May 2025).

This occurs mostly in *Acinetobacter baumannii* with mutations in *PmrB*, a sensor kinase within the *PhoP-PhoQ* regulatory system (Figure 4A). A total of 57 amino acid substitutions were identified in the five functional critical regions, such as *HATPase*, *HAMP*, *TM1*, *TM2*, and *PAS* domain (PD) (Figure 4B) [62]. These changes include substitutions, deletions, and frame shifts that diminish the protein’s ability to regulate its signaling pathways properly, which results in colistin resistance. Key amino acids include N353Y and S403F in the HATPase domain, R165S and P190S in the HAMP domain, M145I and L153F in the TM1/TM2 domains, and L9_G12del and F65L in the PD domain [63]. That such diverse types of mutations are possible in each domain is a testimony to the adaptability of *Acinetobacter baumannii* in acquiring resistance. The genomic epidemiology of colistin-resistant *Acinetobacter baumannii* has identified considerable global diversity represented by 24 STs distributed across five continents [64]. In Asia, the dominant sequence type is ST130, while in Europe, there is the highest diversity, with 10 major STs that include ST195, ST345, ST490, and ST1421 [65]. Dominant STs in South America include ST15, ST25, ST79, and ST730, while ST46 and ST94 are common in North America [66]. The most frequently reported sequence type in Africa was ST158 (Figure 4C). This geographic variation underlines the adaptability of *Acinetobacter baumannii* and the wide dissemination of colistin resistance worldwide [67]. A total of 57 distinct amino acid mutations have been identified across five critical functional domains: *HATPase*, *HAMP*, *TM1*, *TM2*, and the Per-Arnt-Sim (PAS) domain (PD) [68,69].

### 4.3. Chromosomal Genes Lead to Intrinsic Resistance

Resistance occurring naturally to polymyxins is associated with the constitutive expression of the chromosomal *eptB* gene or *arnBCAFTEF* operon [70]. It leads to the addition of *L-Ara4N* or PEtN cationic groups to the LPS in *Serratia marcescens* and *Proteus mirabilis*. These changes raise the total charge of the lipoprotein, which is the primary target of polymyxins. This promotes resistance and decreases polymyxin binding activity. In a study performed by Elizabeth et al., it was found that the expression of the *eptB* gene was further enhanced in colistin-resistant *Escherichia coli* strains after exposure to colistin harboring the *mcr-1* gene [71].

### 4.4. Role of Efflux Pumps in Colistin Resistance

There are many mechanisms that have been involved in the development of colistin resistance. Efflux pumps were found to be among them, yet their role is not fully understood. The EmrAB efflux pumps have been linked to colistin resistance in *Acinetobacter baumannii*, according to research by Ding et al. [72]. A significant decrease in the MICs of resistant strains (by 128- to 512-fold) and complete or partial inhibition of the growth of resistant subpopulations were observed when low doses of the efflux pump inhibitor carbonyl cyanide m-chlorophenylhydrazone (CCCP) were added [73]. Still, due to the non-specific effects of CCPs on the efflux systems and having a broader impact on bacterial metabolism, these findings should be interpreted cautiously [74]. To restore the sensitivity of colistin in colistin-resistant *Escherichia coli* strains, the combined use of an efflux pump inhibitor, which reduces the extrusion of colistin, and a MarR inhibitor, which is known to enhance the binding of colistin, has been presented as a successful strategy both in vivo and in vitro [75].

### 4.5. Plasmid-Associated Colistin Resistance

Plasmids are extra-chromosomal circular DNA molecules found in many species of bacteria, archaea, and even eukaryotes. They can carry a large number of accessory genes encoding various traits, such as regulating bacterial metabolism, colonization, and environmental adaptation. As such, plasmids serve as both an important source of bacterial genetic material and a driving force behind rapid bacterial evolution [46]. The ability of plasmids to horizontally transfer these accessory genes to new hosts can have significant implications for ecosystems and human health, most notably demonstrated by the rapid spread of plasmid-mediated antibiotic resistance genes. The dissemination of MDR conjugative plasmids carrying antibiotic resistance genes has emerged as one of the major public health threats of the 21st century [76]. Plasmids such as IncX4, IncI2, and IncHI2 are typical mobilizable plasmids and serve as key vectors facilitating the rapid spread of *mcr-1* [77]. Additionally, different plasmids can undergo fusion or dissociation through different mechanisms, like plasmid recombination, further driving plasmid evolution. This process enhances plasmid diversity, expands host range, and promotes broader dissemination of resistance genes, such as *mcr-1*. For instance, Sun et al. reported an IS*26*-mediated co-integrate plasmid of the IncX3/IncX4 type carrying both *mcr-1* and *bla*_NDM-5_ [27], while Wu et al. documented an IS*26*-mediated co-integrate plasmid formed by an *mcr-1*-bearing phage-like plasmid and an IncF53:A-:B- plasmid [78,79].

### 4.6. Role of Biofilms in Colistin Resistance

There are different studies that have demonstrated the correlation between colistin resistance in bacteria and the ability to form biofilms, which highlighted their functions in the resistant phenotype [80]. There are different bacterial species that are known to produce biofilms, enhancing tolerance to antimicrobials and impeding their penetration. A recent study performed by Hamel et al. [81] found that there are potential relationships between antibiotic-resistant phenotypes and biofilm formation in clinical *Acinetobacter baumannii* [81]. There are certain key virulence factors that are responsible for biofilm formation, including fimbriae, surface proteins, flagella, pili, and the production of acyl-homoserine lactone (AHL) and poly-β-(1-6)-N-acetylglucosamine (PNAG) signal molecules [82].

A recent proteomics-based study on colistin-resistant *Escherichia coli* revealed the underlying mechanisms of resistance by identifying a panel of differentially expressed proteins. It was found by the study that these proteins and their linked pathways could serve as targets for the development of novel therapies against colistin-resistant infections. Changes in the negative regulator *mgrB* in resistant strains may be associated with elevated expression of quorum-sensing and biofilm-forming genes. Mutations in *mgrB* can cause dysfunction of the *PhoP-PhoQ* two-component system that was responsible for colistin resistance through the cumulative expression of quorum-sensing and biofilm-forming genes [83].

### 4.7. De Novo Gene Evolution Leads to Colistin Resistance

After the discovery of *mcr* genes, these resistance genes have been identified in colistin-resistant isolates globally. They threaten the efficiency of the last-resort antibiotic, i.e., colistin [84]. Broeils et al. reported that new genes can also originate de novo; for example, the human gene CLLU1, expressed in chronic lymphocytic leukemia, arose from previously non-coding sequences [85]. Similarly, Peng et al. demonstrated that *Drosophila* species harbor multiple de novo genes from non-coding DNA that rapidly acquired transcriptional activity [86]. These cases highlight the fact that de novo gene origination is a continued evolutionary process alongside traditional mechanisms, like duplication–divergence.

## 5. Colistin Resistance: A “One Health” Challenge

Antimicrobial resistance, having complex epidemiology, is a public health challenge suited best for in-depth study within the “One Health” framework [87,88]. “One Health” is defined as the combined effort of various disciplines that are working together at national, local, and global levels to achieve optimal health outcomes for animals, people, and the environment through practice, policy, research, and education [89]. The use and even misuse of antimicrobials in animals, humans, and the environment, and the subsequent spread of resistant bacteria across these sectors around the world, serve as primary drivers of antimicrobial resistance [90,91]. One major problem in the field of animal health and agriculture is the overuse of antimicrobials that are vital to human health, including colistin, which is used as a last option to treat Gram-negative bacterial infections. Uncontrolled usage of colistin in both veterinary medicine and humans has been found to be responsible for the emergence of colistin resistance in *Enterobacteriaceae* [92]. Colistin is a polymyxin last-resort antibiotic and has recovered clinical relevance in the treatment of multidrug-resistant Gram-negative bacteria [93]. However, since the 2015 emergence of the plasmid-borne *mcr-1* gene that is horizontally transmissible and initially found in swine farms, it has rapidly disseminated among humans, livestock, foodstuffs, and the environment [87]. Further, *mcr* variants (*mcr-1* to *mcr-10*) have appeared worldwide, commonly in conjunction with other resistance genes, therefore multiplying public health hazards [56]. Surveillance studies using the One Health approach, including the characterization of colistin-resistant Enterobacterales in human healthcare institutions, livestock, and food supplies in the Netherlands and Belgium, have disclosed extensive prevalence throughout all sectors as well as a significant correlation between the usage of colistin as a farm growth promoter with rates of resistance [30]. A successful decrease in colistin-resistant *Escherichia coli* since withdrawal of colistin as a farm growth promoter provides the best example of the success of cross-sector coordinated stewardship policies [94]. Individually, these studies emphasize that to combat colistin resistance, we need to adopt a collaborative, multi-disciplinary One Health approach combining surveillance, regulation, and stewardship in all human, animal, and environmental sectors [31,61].

### 5.1. Colistin Resistance in Clinical Settings

International consensus recommendations offer guidance on colistin therapy and its ideal clinical application [93]. For MDR Gram-negative bacteria, like *Pseudomonas* species, *Acinetobacter*, and carbapenemase-producing Enterobacterales, colistin is a last-line defense [95,96]. In intensive care units (ICUs), it is mainly used in critical conditions, like pneumonia and sepsis/bacteremia linked with ventilator-associated pneumonia (VAP). In other cases, colistin has been viewed as an alternative treatment for conditions like osteomyelitis, meningitis, soft tissue infections, gastrointestinal infections, urinary tract infections, joint infections, eye and ear infections, and pyoderma [97]. As it is known to have nephrotoxicity, colistin must be administered with caution, including close monitoring of patients with renal impairment and dose adjustments [98]. The use of colistin in combination with other antibiotics like amikacin, rifampicin, and ceftazidime has resulted in synergistic effects mainly for treating infections caused by MDR *Pseudomonas aeruginosa* [99].

A number of clinical isolates being resistant to colistin have also been shown to be resistant to a broad spectrum of antimicrobial agents, including carbapenems, nitrofurans, penicillins, quinolones, monobactams, and aminoglycosides [100]. *Klebsiella pneumoniae* from Pakistan was resistant to colistin and was resistant to twenty-four antimicrobial agents across nine different groups, with the exception of tigecycline [101]. Similarly, the prevalence of *mcr-1* isolated from *Salmonella* was found to be 2% in children who were suffering from diarrhea and were under five years old. It highlighted the potential risk of multidrug-resistant strains of *Salmonella* that harbored *mcr-1*, posing a potential threat to public health [102,103]. In a recent study, Portes et al. reported that in Salmonella Typhimurium, the newly identified *mcr-9* gene, found in a clinical isolate in the USA, conferred phenotypic resistance to colistin in *Enterobacteriaceae*, thereby raising significant public health concerns [51]. They involved some factors responsible for the spread of colistin resistance, such as the transportation of food, global travel, and trade to countries having an unknown or high prevalence, like Canada [56], Japan [104], and the USA [60], and in Argentina, there was an over-prescription of colistin to treat highly resistant clinical pathogens in human medicine.

### 5.2. Colistin Resistance in Veterinary and Agriculture Settings

Colistin is being used nowadays as an antibiotic in veterinary medicine majorly in pigs to treat intestinal infections by Enterobacterales orally [105]. To control intestinal infections, it is employed orally in food-producing animals, like pigs and poultry. It has been found by different researchers that compared to untreated pigs, in the pigs treated with colistin, a greater proportion of resistant bacterial isolates were found [106]. Colistin sulfate is used in the seafood sector to encourage fish development, in addition to being used in pigs and poultry. In veterinary settings, the widespread use of colistin places strong selective pressure on animals and practitioners [107]. Gastrointestinal diseases caused by Gram-negative bacteria in calves are treated by oral administration of colistin. There are certain pharmaceutical formulations present that provide significant combined therapy in the sulfate form of colistin, despite being used as monotherapy in oral treatments [108].

Colistin is most frequently used with other antimicrobials, including beta-lactams and amoxicillin, representing its primary example [109]. If colistin is used frequently, it significantly contributes to the development of bacterial resistance and leads to the emergence and spread of *mcr-1* to *mcr-10* genes among the microbial community [110]. A study was conducted by Lu et al., and they reported a high prevalence of 54.6% *mcr-1*-positive *Escharchia coli* from pig feces in Sichuan Province before the colistin ban [111]. Isolates obtained from animals showed an increased rate of resistance against colistin compared to clinical isolates. *Escherichia coli* isolates that were recovered from milk were found to be resistant to colistin [112]. Liu. Y. et al. examined mobilized colistin resistance (*mcr*) genes that have been found in six continents, including North America, Oceania, Asia, South America, Africa, and Europe, and in more than 26 bacterial species since the discovery of *mcr-1* in China [22]. Due to the prolonged use of polymyxins, including colistin, in veterinary medicine, the number of reported cases has increased rapidly.

The prevalence of *mcr-1*-positive *Escherichia coli* rose sharply, reaching 30% by 2014, which correlated with the massive annual use of colistin (2470–2875 metric tons) in livestock. These findings strongly suggest that colistin use in food-producing animals has driven the emergence and expansion of *mcr-1*, underscoring the urgent need for stewardship policies and restrictions on agricultural antibiotic use to curb resistance [113].

## 6. Molecular and Genomics Techniques in Detecting Colistin Resistance

### 6.1. Molecular Detection for the MCR Family

The microarray technique evaluated by Bernasconi et al. [114] has the ability to simultaneously detect both *mcr-1* and *mcr-2* genes and β-lactamases from the bacterial culture. One of the significant advantages of this technology is that it can be upgraded with emerging or new resistance genes for the detection of more types of *mcr* genes and their variants [115]. It requires almost 6.4 h for detection through a microarray with a cost range of 60 to 90 EUR. But, the CT103XL microarray system designed by Gogry et al. is not capable for the detection of variants of the *mcr* gene, including *mcr-3* and *mcr-4* [27]. Although it has certain limitations, it is still very helpful in the detection of emerging antibacterial resistance genes. Its application is hindered due to its rising cost and the requirement of specialized expertise for its operation. Imirzalioglu et al. [116] evaluated a LAMP instrument that is commercially available, labeled eazyplex^®^ SuperBug, for the detection of *mcr-1* from the cultured bacteria, having a turnaround time of about 20 min.

The TaqMan probe-based assay was developed for the detection of the *mcr-1* gene by Chabou et al. using probes and primers that were designed by themselves [117]. eal-time PCR is a significant technique for the identification of genetic markers present in bacteria, providing quick results as shown in Table 2. Another advantage of this PCR technique is that it eliminates the requirement of risky agents, like ethidium bromide [118]. Conventional PCR can detect only one type of *mcr* gene, while real-time and multiplex PCR perform well in the detection of *mcr* genes and their variants in a single assay. Tolosi et al. extended the use of real-time PCR to include the *mcr-1*, *mcr-2*, and *mcr-3* genes for the detection of the *mcr-4* and *mcr-5* genes in both bacterial isolates and environmental samples [119,120].

**Table 2:** Primer sequences used for PCR detection of *mcr* genes (*mcr-1* to *mcr-10*) and these primers were incorporated from and designed by [121].

Serial No.	Primer Name	Primer Sequence (PCR)	Amplicon Size (bp)	Target Gene
**1**	*mcr-1F*	AGTCCGTTTGTTCTTGTGGC	320	*mcr-1*
	*mcr-1R*	AGATCCTTGGTC TCGGCTTG		
**2**	*mcr-2F*	CAAGTGTGTTGGTGCGAGTT	715	*mcr-2*
	*mcr-2R*	TCTAGGCCGACAAGCATACC		
**3**	*mcr-3F*	AATAAAAATTGTTCCGCTTAG	929	*mcr-3*
	*mcr-3R*	AATCGCACATCCCCGTTTT		
**4**	*mcr-4F*	TCACTTTCATCACTGGGTTG	1116	*mcr-4*
	*mcr-4R*	TTGGTCATCGACTACCAATG		
**5**	*mcr-5F*	ATGCCGTTGCTGCCATTTATC	1644	*mcr-5*
	*mcr-5R*	TCATTTGCGTTGGTCTTTC		
**6**	*mcr-6F-mp*	AGCTATGTCAATCCCGTGAT	252	*mcr-6*
	*mcr-6R-mp*	ATTGGCCTAGGTTGCATC		
**7**	*mcr-7F-mp*	GCCCTTCTTTTCGTTGTT	551	*mcr-7*
	*mcr-7R-mp*	GGTTGCTCTCTTTCTCGGT		
**8**	*mcr-8F-mp*	TCAACAATTCTACAAAGCGTG	856	*mcr-8*
	*mcr-8R-mp*	AGTTTGGGTCTAAAGAGG		
**9**	*mcr-9F-mp*	TTCCCTTTGTTCTGGTGTTG	1011	*mcr-9*
	*mcr-9R-mp*	GCAGGTAATAAGTCGGTC		
**10**	*mcr-10F*	AGCCGTCTTGAACATGTGAG	744	*mcr-10*
	*mcr-10R*	CATACAGGGCACCCGAGACTG		

A quantitative TaqMan^®^ PCR assay was developed by Gong et al., building on the work of Liu et al., for the qualitative and quantitative detection of *mcr-1* genes in chicken feces and cultured bacteria [119]. By utilizing multiplex PCR, two recent molecular assays were developed by Li et al. to detect *mcr-1* to *mcr-5*, which was followed by agarose gel electrophoresis (1.6% and 2.7%, respectively) and ethidium bromide staining [122]. A SYBR green real-time PCR method was used by Badr et al. to increase *mcr-1* detection in stool samples from humans. The main contribution of the research was demonstrating that the enrichment of stools in LB broth supplemented with colistin significantly increased the detection rate over examination of indigenous stools [123]. Ali et al.’s study focused on the development of a single real-time PCR method for the identification of *mcr-1* and *mcr-2* in *Escherichia coli* isolated from fecal samples [124].

### 6.2. Genomics and Sequencing

Genomic techniques occupy an essential site to expand the molecular processes behind colistin resistance and monitor its worldwide dissemination. For the detection and characterization of antimicrobial resistance and genes conferring resistance, like *mcr*, next generation sequencing (NGS) has been found to be an effective tool with a high-throughput advantage [21]. For the genomic profiling of bacterial pathogens, it can deliver genomic data covering all resistance genes and other CDS effectively in a single analysis. Bacterial clonal groups involved in public health, as well as the molecular characterization of epidemic plasmids carrying virulence or AMR genes, can be effectively studied using NGS, which serves as a powerful tool [125]. To analyze the entire genome, whole-genome sequencing (WGS) is used in the detection of novel and already known resistance mechanisms. Based on the equipment used, it can detect these mechanisms within a few days and even in real-time [126]. The first plasmid-mediated gene, *mcr-1*, was identified through WGS [127]. These resistance determinants include plasmid-borne *mcr* alleles and chromosomal alterations within regulatory modules, like *pmrA/pmrB* and *mgrB* [84].

Comparative genomics extends these understandings by aligning resistant and susceptible isolates to identify genetic dissimilarities underlying resistance. It simultaneously exposes the role of mobile genetic constituents in transmitting resistance attributes [128]. Concurrently, PCR and qPCR continue to be indispensable for swift, specific-gene detection. They also provide economical strategies for clinical diagnostics and ecological surveillance. Metagenomic sequencing enhances these approaches by analyzing DNA from entire microbial consortia without cultivation. This enables researchers to evaluate resistance reservoirs in multifaceted ecosystems such as wastewater, soil, and animal microbiota [129]. Beyond identification, functional and expression-oriented genomic techniques are essential for interpreting resistance at the molecular dimension. Transcriptomic profiling, such as RNA sequencing (RNA-seq), discloses transcriptional modifications induced by colistin exposure. It clarifies regulatory circuits that remodel bacterial outer-membrane architecture and diminish drug affinity [130]. Functional genomics, such as CRISPR-Cas-mediated editing and directed gene knockout analyses, substantiates the contributions of candidate genes. It directly connects genotypes to resistance phenotypes. The integration of these approaches offers a holistic representation of the genetic framework of colistin resistance. It also provides a scaffold to evaluate evolutionary restraints, detect emergent threats, and guide antimicrobial stewardship policies [131]. Together, these genomic techniques establish a scientific foundation for both surveillance and the development of interventions aimed at limiting the dissemination of colistin resistance.

## 7. Antibiotics to Tackle the Colistin-Resistant Bacterial Infections

### 7.1. Antibiotic Monotherapy for Colistin-Resistant Pathogens

Petrosillo et al. [132] conducted a study on the phenomenon of colistin resistance in *Klebsiella pneumoniae* and the consequences of this phenomenon for treatment options, since antimicrobials such as tigecycline, gentamicin, fosfomycin, and ceftazidime/avibactam are often primary candidates and were also ineffective. The authors explained that antimicrobial choice is based on the drug’s pharmacokinetics and pharmacodynamics, site of infection, resistance pattern, and toxicity potential. Petrosillo et al. [132] also address the antimicrobial treatments that are available for colistin-resistant and multidrug-resistant *Klebsiella pneumoniae*. The newly developed drugs or experimental phases, such as plazomicin and cefiderocol, bacteriophages, and monoclonal antibodies, can be used as an alternative treatment for *Klebsiella pneumoniae*, as shown in Table 3.

A study was conducted by Papazachariou et al. [133], and they observed overdosing of intravenous colistin monotherapy in the treatment of multidrug-resistant *Acinetobacter baumannii* infections in critically ill patients. But, using colistin alone was the least effective in the presence of high morbidity and mortality, considering prolonged stay in the ICU and a high mortality rate. Thus, despite the presence of colistin alone as a treatment option, stronger inherent limitations are noted for achieving rapid clinical stabilization and for optimized survival rates. Papazachariou et al.’s findings were also indicative of no other treatment alternatives in *Acinetobacter baumannii* and the associated need to reduce the treatment outcome, as shown in Table 3.

### 7.2. Antibiotic Combination Therapy for Colistin-Resistant Pathogens

In the international randomized trial OVERCOME by Kaye et al. [134], using both meropenem and colistin was correlated with better clinical and microbiological relief than colistin alone for patients with pneumonia and bloodstream infections due to extensively drug-resistant *Acinetobacter baumannii*, *Pseudomonas aeruginosa*, and *Enterobacterales*, as shown in Figure 1B. Their results also demonstrated improved clinical relief and suggested that future research with the colistin–meropenem combination should be undertaken for Enterobacterales and *Pseudomonas aeruginosa* due to the trends of decreased mortality seen in the OVERCOME and AIDA trials. However, newer antimicrobial compounds were preferred as alternatives based on the documented better safety and efficacy profiles than colistin of contemporary beta-lactam/beta-lactamase inhibitor combinations for these infections [134].

The study conducted by Shein and colleagues [135] on XDR Gram-negative bacteria hotspots found that colistin was almost always combined with carbapenems in an attempt to increase antibacterial effectiveness and reduce the risk of additional resistance development. The growing colistin resistance was clinically concerning because it was the last-resort therapy. Clinically, it was suggested that colistin–carbapenem combination therapy would likely improve outcomes and slow the onset of reduced colistin susceptibility. Consequently, a double-blind randomized controlled trial was conducted in patients with pneumonia and bloodstream infections due to XDR Gram-negative bacteria to measure the effectiveness of combination therapy and to clarify the pharmacokinetic and microbiological factors (synergistic activity, colistin concentration in plasma) that would influence therapeutic response, nephrotoxicity, and outcome, as shown in Table 3 [135].

A case report was published by Rolain and colleagues [136] of a 58-year-old male patient from France with colonization of colistin-resistant *Acinetobacter baumannii* after a prolonged stay in the intensive care unit. Due to severe pneumonia and acute respiratory failure, he needed to be mechanically ventilated and put on a therapy after the use of ampicillin–clavulanate and piperacillin–tazobactam with spiramycin to imipenem and amikacin therapy for acute respiratory distress syndrome and septic shock. While he was on colistin and rifampicin therapy after he was found to have colistin susceptible strain of *Acinetobacter baumannii* in his blood and bronchoalveolar lavage, he clinically stabilized in a manner such that he was taken off of extracorporeal membrane oxygenation. A combination regimen of imipenem, amikacin, and colistin therapy was reintroduced after a septic shock event, and no blood cultures were taken. The patient continued to improve with respiratory function, but he was found to have colistin-resistant *Acinetobacter baumannii* tracheal colonization without the presence of a respiratory infection. Asymptomatic *Acinetobacter baumannii* bacteriuria was found, but the patient was treated with colistin for a time until resistant *Acinetobacter baumannii* were isolated, and the patient was discharged from the intensive care unit [136].

Another case reported from Spain by López-Rojas et al. [137] described a patient who came to the hospital’s neurology department suffering from a colistin-susceptible *Acinetobacter baumannii* infection in the central nervous system and ongoing complications from his previous surgery of craniopharyngioma resection. The patient then developed a CSF infection with a multidrug-resistant and colistin-susceptible strain of *Acinetobacter baumannii*. The antibiotic therapy was switched to colistin, which resulted in the isolation of colistin-resistant *Acinetobacter baumannii* and a chronic infection with purulent meningitis. To address the infection, histotoxic infection, and CSF purulence, the patient was given intrathecal gentamicin. A stepwise decrease in CSF purulence was observed with modifications to the patient’s antibiotic therapy in accordance with the sensitivity to the *Acinetobacter baumannii* strain and the CSF inflammatory response. To account for the persistent hydrocephalus, the patient’s antibiotic regimen was broadened to include IV soft beta-lactams, and gentamicin was discontinued. The patient’s elevated CSF white blood cell and protein count decreased as expected, and with this, the patient was declared infection-free. The case report of López-Rojas et al. demonstrated that high virulence strains of *Acinetobacter baumannii* can acquire colistin resistance alongside other multidrug resistances [137].

Another case reported by Dehbanipour et al. [138] found that the heteroresistance phenotype had subpopulations of bacteria with lesser antibiotic susceptibilities than the dominant population. Routine diagnostic labs had insufficient sensitivity to detect heteroresistance. Detecting heteroresistance by more sophisticated means was both expensive and took a long time. The emergence of heteroresistance to colistin was a genuine public health concern. As of today, heteroresistance to colistin has been documented in *Acinetobacter* spp., *Klebsiella* spp., *Enterobacter* spp., *Pseudomonas* spp., *E. coli*, *Salmonella enterica serovar Typhimurium*, *Neisseria meningitidis*, and *Stenotrophomonas maltophilia*. The growing heteroresistance to colistin was a public health concern and warranted immediate global attention. This was because the mechanisms that drove the emergence of hetero-resistance to colistin in bacteria were useful in formulating ways to reduce resistance and design new treatment options. The analyzed data showed that frequencies differed by country, and that the implementation of tailored treatment techniques, especially those involving multiple medications, along with improved diagnostics, may reduce the emergence of resistant subpopulations and enhance treatment results [138].

## 8. Alternative Therapeutic Strategies for Colistin-Resistant Pathogens

To overcome this issue, the development of new drugs and molecules, combination therapies that involve drugs and colistin, re-purposing existing medications, strategies like photodynamic therapy, phage-based therapies, other innovative strategies like nano-based approaches, and *CRISPR/Cas* are all being investigated to deal with these fatal colistin-resistant pathogens, as shown in Table 3 [139].

### 8.1. Herbal Therapy for Colistin-Resistant Pathogens

Foda and colleagues [140] conducted an in vitro experiment on plant-sourced essential oils (cinnamon, thyme, and eucalyptus) as alternative antimicrobial agents for colistin-resistant pathogens. For the study, bacterial isolates were obtained from hospitalized patients, and the selected essential oils were evaluated for antimicrobial activity, possible cytotoxicity, and composition. From the oils tested, cinnamon was the strongest against all colistin-resistant isolates, and thyme and eucalyptus oil were the next highest. The activity of cinnamon oil was the strongest against all colistin-resistant isolates, and it was followed by thyme oil. The exposure of elms to cinnamon oil was the strongest against all colistin-resistant isolates. Cinnamon oil activity was also destructively impacted on bacterial viability, and the *mcr-1* gene expression was primarily silenced. Foda and colleagues’ findings indicated that cinnamon oil, as well as other plant-derived compounds, offered considerable antibioactivity for colistin-resistant bacteria and may be of value as an additional or alternative therapeutic for the treatment of advanced infections caused by colistin Gram-negative bacteria [140].

Liu et al. [141] studied the effective antimicrobial strategy of colistin combined with the Chinese herbal medicine shikonin against colistin-resistant *Escherichia coli.* Their experimental outcomes showed that shikonin significantly enhanced the antibacterial efficacy of colistin against colistin-resistant *Escherichia coli*. In vitro combination therapy reduced colistin minimum inhibitory concentration, improved bactericidal activity, and improved survival in the *Galleria mellonella* infection model. The colistin–shikonin representation decreased the biofilm formed by the bacteria through the downregulation of key genes involved in biofilm formation by membrane disruption of the bacteria. Their mechanistic studies showed the synergy between colistin and shikonin stems from elevated reactive oxygen species and the *mcr-1* gene suppression. Most importantly, Liu et al.’s work suggested that shikonin is a promising colistin adjuvant and, together, provides an effective strategy to address infections caused by colistin-resistant *Escherichia coli* [141].

Researchers are struggling to find new drugs and molecules having novel modes of action so that they can be effectively used against a range of MDR species [142]. Terrein, which is a refined compound derived from fungus, has exhibited antimicrobial effects against *Aeromonas hydrophila*, *Staphylococcus aureus*, *Enterococcus faecalis*, and some other microbes [143]. In the same way, some Fluopsin C, which is a bioactive secondary compound composed of metal extracted from *Pseudomonas* and *Streptomyces* species, has been known to show considerable antimicrobial activity against Gram-negative, Gram-positive, and drug-resistant bacteria [142]. According to this, following clinical approvals, alternative strategies such as probiotics, organic acids, trace minerals, immune modulators, antimicrobial peptides, vaccines, prebiotics, and gene editing technologies (Cas9) provide a diverse set of tools to combat colistin-resistant bacteria. Following clinical validation, many of these molecules could serve as potential replacements for colistin in both prevention and treatment settings.

### 8.2. CRISPR/Cas9 for Colistin-Resistant Pathogens

The study conducted by Wan et al. focused on a *CRISPR-Cas9* strategy via plasmids to remove the *mcr-1* gene and recover colistin sensitivity in *Escherichia coli*. From the mcr-1 gene, two specific sgRNAs were designed, and mcr-1-bearing plasmids were shown to be cured both in the plasmid-modifying and in the naturally occurring case. This was verified by the authors using the PCR and the qPCR analysis of the mcr-1 plasmids. The authors state that of the two factors of composition of the backbone of the plasmids and the length of the sgRNA, the backbone composition was the most important. Using a *CRISPR-Cas9* approach to prevent the horizontal transfer of the *mcr-1* gene to other cells was also demonstrated. The results indicate that this *CRISPR-Cas9* approach is the first to demonstrate that *Escherichia coli* can be re-sensitized to colistin and to also show the first results in potentially limiting the spread of colistin resistance mediated by plasmids, as shown in Table 3 [144].

Alkompoz and colleagues examined the occurrence of the *CRISPR/Cas* system in clinical isolates of Klebsiella pneumoniae from Egypt, as well as its antimicrobial susceptibility and how it compares with the available genomes worldwide. Screening 181 clinical isolates by PCR and performing an in silico analysis of 888 complete genomes confirmed that *CRISPR/Cas* systems were present in about 25% of the strains, most of which were of types I-E and I-E*. Although the majority of the strains had multidrug-resistant and extensively drug-resistant (MDR/XDR) phenotypes, *CRISPR/Cas*-positive and -negative strains did not differ in terms of antimicrobial susceptibility/resistance or the number of resistance genes. The genomes that did not have *CRISPR/Cas* systems had a comparatively higher number of resistance genes and plasmids, but these differences did not hold any significant value in spacer content, which showed a weak correlation with resistance determinants. The conclusion that *CRISPR/Cas* systems did not influence the antimicrobial resistance profiles signified the presence of a low level of natural targeting of resistance plasmids and the value of conducting more targeted analyses of spacers and plasmids [145].

### 8.3. Phage Therapy for Colistin-Resistant Pathogens

Phage therapy has emerged as a promising tool for combatting antibiotic resistance and bacterial infection. Rahimian et al. found that, at pH 7, phage particles were found to carry a negative charge; on the other hand, colistin-resistant bacteria were found to have less negative zeta potentials compared to the wild type [146]. As colistin-resistant cells have a lower negative surface charge, there was less electrostatic repulsion between the bacteria and the phage, which promotes phage adhesion and infection [147]. In another study, it was found that the combination of colistin with the phage Phab24 changed the enveloped structure of colistin-resistant *Acineobacter buamannii*, causing a reduction in resistance. This reduction in antibiotic resistance was found to be directly linked with the phage-resistance mechanism that can be applied in clinical settings [148].

Xiao et al. [149] conducted a study that focused on phage therapy as an alternative solution to the problem of multidrug-resistant *Escherichia coli*, including colistin-resistant *Escherichia coli*. A total of 52 bacteriophages were isolated from livestock farm samples in four different provinces in China. One of these samples, pig feces, yielded the novel bacteriophage *vB_EcoStr-FJ63A*. Morphological and genomic characterization indicated that this phage has an icosahedral capsid, an inflexible tail, and a large double-stranded DNA genome. Taxonomically, this phage was classified as a *Krischvirus* in the family of *Straboviridae*. This phage exhibited high stability across a large range of temperatures and pH, as well as lytic activity against many different colistin-resistant *Escherichia coli* from different animals. It also exhibited no virulence or resistance genes that would make it less than ideal for phage biocontrol. These characteristics make *vB_EcoStr-FJ63A* an ideal candidate for colistin-resistant *Escherichia coli* biocontrol3 [149].

### 8.4. Probiotics for Colistin-Resistant Pathogens

Das and colleagues evaluated human gut-derived probiotics as a potential prophylactic approach for addressing the therapeutic challenges associated with multidrug- and colistin-resistant *Klebsiella pneumoniae*. Probiotic *Lacticaseibacillus* isolates were assessed for their ability to survive gastric conditions and were able to inhibit pathogenic *Klebsiella pneumoniae* in vitro, which was due to the presence of organic acids and a heat-labile protein/amino acid antimicrobial factor in the cell-free supernatant. Of the two *Lacticaseibacillus* paracasei isolates chosen for further study based on their antimicrobial activity and adhesion to CaCo-2 cells, one demonstrated more consistent ultrastructural damage to the treated pathogens by scanning electron microscopy. Both live and heat-inactivated formulations of the selected probiotics reduced the microbiological burden and histopathological damage more than the untreated control in a Balb/c mouse model. Whole-genome sequencing proved the absence of antibiotic resistance and virulence factors. Bioinformatics and whole-genome sequencing predicted the presence of secreted antimicrobial factors, providing further evidence for the use of these probiotics as safe and effective prophylactic agents against multidrug-resistant *Klebsiella pneumoniae* [150,151].

**Table 3:** Antibiotic and alternative therapeutic strategies against colistin-resistant Gram-negative bacteria.

References	Bacterial Strain (Colistin-Resistant)	Resistance Mechanism/Phenotype	Type of Therapy	Study Design/Model	Study Outcomes
[132]	*Klebsiella pneumoniae*	Colistin resistance (clinical CRKP)	Colistin monotherapy	Narrative review	Combination therapy and non-antibiotic strategies are required
[133]	*Acinetobacter baumannii*	Colistin resistance	Colistin-based combinations	Narrative review	Microbiological cure; monotherapy inadequate for Col-R *Acinetobacter baumannii*
[132]	*K. pneumoniae*, *Acinetobacter baumannii*, *Pseudomonas aeruginosa*	Colistin-resistant CROs	Combination therapy	Meta-analysis	Mortality reduction; combination therapy significantly reduced mortality
[134]	*Klebsiella pneumoniae*	*mgrB*-mediated resistance, biofilm	Colistin + EDTA	In vitro	MIC reduction, biofilm disruption, EDTA restored colistin activity
[152]	*Klebsiella pneumoniae*	Colistin resistance	Combination regimens	Systematic review and meta-analysis	Clinical cure and combination therapy are superior in bloodstream infections
[138]	Gram-negative bacteria	Colistin heteroresistance	Diagnostic and therapeutic strategies	Review	Detection and treatment efficacy and heteroresistance are underdiagnosed
[140]	*Escherichia coli*, *Klebsiella pneumoniae*, *Pseudomonas aeruginosa*	Colistin resistance	Essential oils	In vitro	MIC inhibition and natural compounds show adjunct potential
[141]	*Escherichia coli*	mcr-mediated resistance	Colistin + shikonin	In vitro and in vivo	Synergistic killing restored susceptibility
[144]	*Escherichia coli*	*mcr-1* plasmid	*CRISPR-Cas9*	In vitro	Resistance reversal and gene editing eliminated colistin resistance
[149]	*Escherichia coli*	Colistin-resistant zoonotic strains	Lytic bacteriophage	In vitro and in vivo	Bacterial lysis, survival, and phages were effective against Col-R isolates

## 9. International Policy Interventions and Control of Colistin Resistance

**Figure 5:**
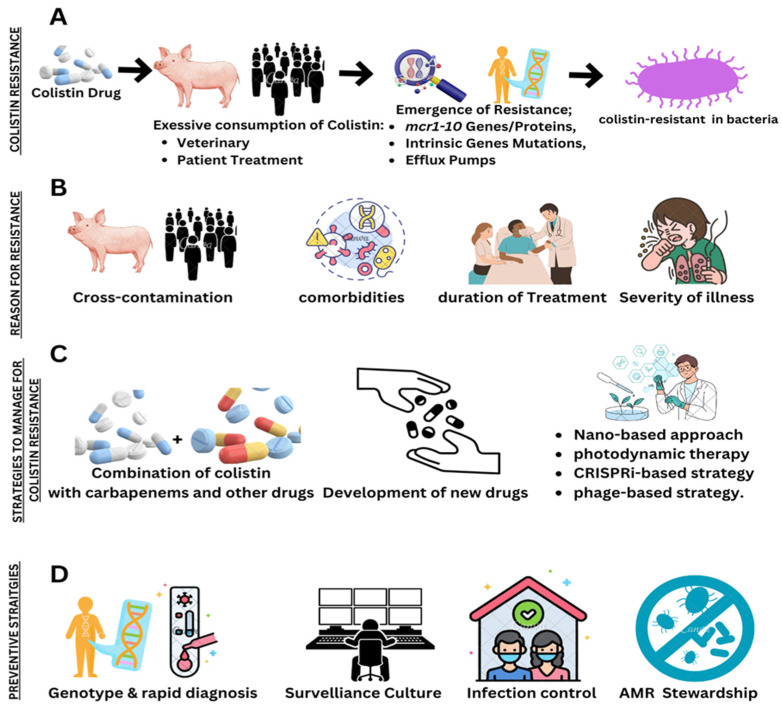
(**A**) Development of colistin resistance. (**B**) Reasons for colistin resistance. (**C**) Proposed strategies to tackle colistin resistance. (**D**) Preventive measures for colistin resistance. (Created in https://BioRender.com, accessed on 10 June 2025).

The ban on colistin use as a growth promoter in farm animals has led to a reduction in colistin-resistant bacteria, including resistance determinants, such as the *mcr-1* gene, in humans, animals, and the environment [153]. The prevalence of colistin resistance poses a significant threat to the health of humans, animals, and the environment, as shown in Figure 5. The widespread use of colistin has contributed to the dissemination of *mcr* genes in bacteria originating from humans and food-producing animals [154]. The key driver for positively increasing the prevalence of *mcr-1*-carrying bacteria is the heavy use of colistin in the food-producing animal industry. In China, after the initial identification of *mcr-1* in 2017, authorities prohibited the use of colistin as a feed additive for livestock. Inspections have recorded a marked decline in the frequency of colistin-resistant *Escherichia coli* “*mcr-1*”among both animals and humans in China following this prohibition. For example, one study reported that *mcr-1* occurrence in swine feces decreased from 45% in 2016 to 19% in 2018 [153].

In Vietnam, the government has adopted a strategic plan to gradually eliminate the preventive administration of antibiotics, including colistin, with a complete ban on prophylactic application in livestock set for 1 January 2026 [155]. India likewise took an important measure by outlawing the manufacture, marketing, and distribution of colistin and its formulations for food-producing animals in July 2019. This decision was driven by evidence of the widespread misapplication of colistin as a growth enhancer in the Indian poultry sector [156]. The European Union, while not imposing a total ban on veterinary use, has established stringent directives. These include a universal ban on antibiotics for growth stimulation, the mandatory requirement of a veterinary prescription, and classification frameworks that restrict the use of critically important antimicrobials, such as colistin [105].

To manage and curb the dissemination of colistin-resistant bacteria, robust antimicrobial governance and strict infection containment strategies are essential. This entails limiting colistin application exclusively to verified MDR infections and enforcing isolation precautions for colonized or infected patients to block intra-hospital transmission [157]. Routine and meticulous sanitization of healthcare environments with suitable disinfecting agents, together with rigorous hand hygiene compliance by medical personnel, are pivotal in interrupting the transmission cycle. Educating staff on the hazards of antimicrobial resistance further enhances adherence to infection prevention standards. The European Union and South Korea serve as examples where stringent stewardship initiatives have demonstrated success in mitigating resistance. Nonetheless, the increasing prevalence of resistance, as documented by [158], underscores the urgency of implementing comprehensive stewardship frameworks in clinical contexts [159].

Clinical examinations indicated that the colistin used in tandem with tigecycline and gentamicin has been correlated with reduced mortality rates in MDR *Klebsiella pneumoniae* infections [132]. Beyond traditional drugs, investigators are examining antimicrobial peptides, nanomaterials, and therapeutic adjuvants to enhance colistin’s efficacy against resistant strains. These innovative interventions hold promise for restoring bacterial susceptibility and decreasing the therapeutic levels of colistin required for effective treatment for *Acinetobacter baumannii* [160].

## 10. Conclusions and Future Perspectives

Colistin resistance has emerged as a challenging issue in treating bacterial infections. As many studies reported, there is a worldwide distribution of colistin resistance genes (*mcr-1* to *mcr-10*) that play an important role in AMR crises. The reported prevalence of colistin resistance throughout the world has been documented in numerous bacterial species and different sources, which indicates their potential transmission across species and geographical borders. The *mcr-1*–*10* genes are key contributors in developing colistin resistance among different bacterial species. We need specific and wide actions to handle the situation, including strict regulation of its use, routine surveillance, and intervention strategies. Intervention strategies may include combination treatment with colistin and other drugs, drug re-purposing, and some novel approaches, such as phage therapy and photodynamic therapy, which have been developed to combat resistance against colistin. The development of various novel approaches comprising new antimicrobial compounds and advanced techniques, like CRISPR, may provide grounds for optimism in addressing this increasingly dire challenge of colistin resistance. Eventually, each one constitutes a very important piece in the broad puzzle of how to handle the problem of MDR pathogens, ensuring further effectiveness of treatments to the last-line antibiotics, such as colistin. We need to focus on the development of new therapeutic strategies to overcome colistin resistance and its misuse in humans and animals. Additionally, researchers have to move on to the development of antibiotics and novel molecules having enhanced efficacy and low toxicity in comparison to colistin. This includes exploring non-antibiotics and alternatives to colistin and the use of antibiotics in combinations, along with the development of antibiotic synergists.

## Data Availability

The original contributions presented in this study are included in the article. Further inquiries can be directed to the corresponding authors.

## Abbreviations

The following abbreviations are used in this manuscript:

MDR	Multidrug resistance
MBLs	Metallo-beta-lactamases
NDM	New-Delhi MBL
CS	Colistin sulfate
CMS	Colistin methanesulfonate
AMR	Antimicrobial resistance
LPS	Lipopolysaccharide
CyD	Cytochrome D
VAP	Ventilator-associated pneumonia
MIC	Minimum inhibitory concentration
PD	*PAS* domain
CCCP	Carbonyl cyanide m-chlorophenylhydrazone
AHL	Acyl-homoserine lactone
PNAG	Poly-β-(1-6)-N-acetylglucosamine
NGS	Next generation sequencing
WGS	Whole-genome sequencing
MLST	Multi-locus sequence typing
PDT	Photodynamic therapy
